# Supplementary material for: Effectiveness of BNT162b2 after extending the primary series dosing interval in children and adolescents aged 5–17
Source: Nat Commun. 2023 Apr 3;14:1845. doi: 10.1038/s41467-023-37556-z (PMC10068718; doi:10.1038/s41467-023-37556-z)
Supplement: Supplementary file 1 — Supplementary Information [file 41467_2023_37556_MOESM1_ESM.pdf]

Title: *Effectiveness of BNT162b2 after extending the primary-series dosing interval in children and adolescents aged 5-17*
Journal: *Nature Communications*

Supplementary Figure. Sample size calculation

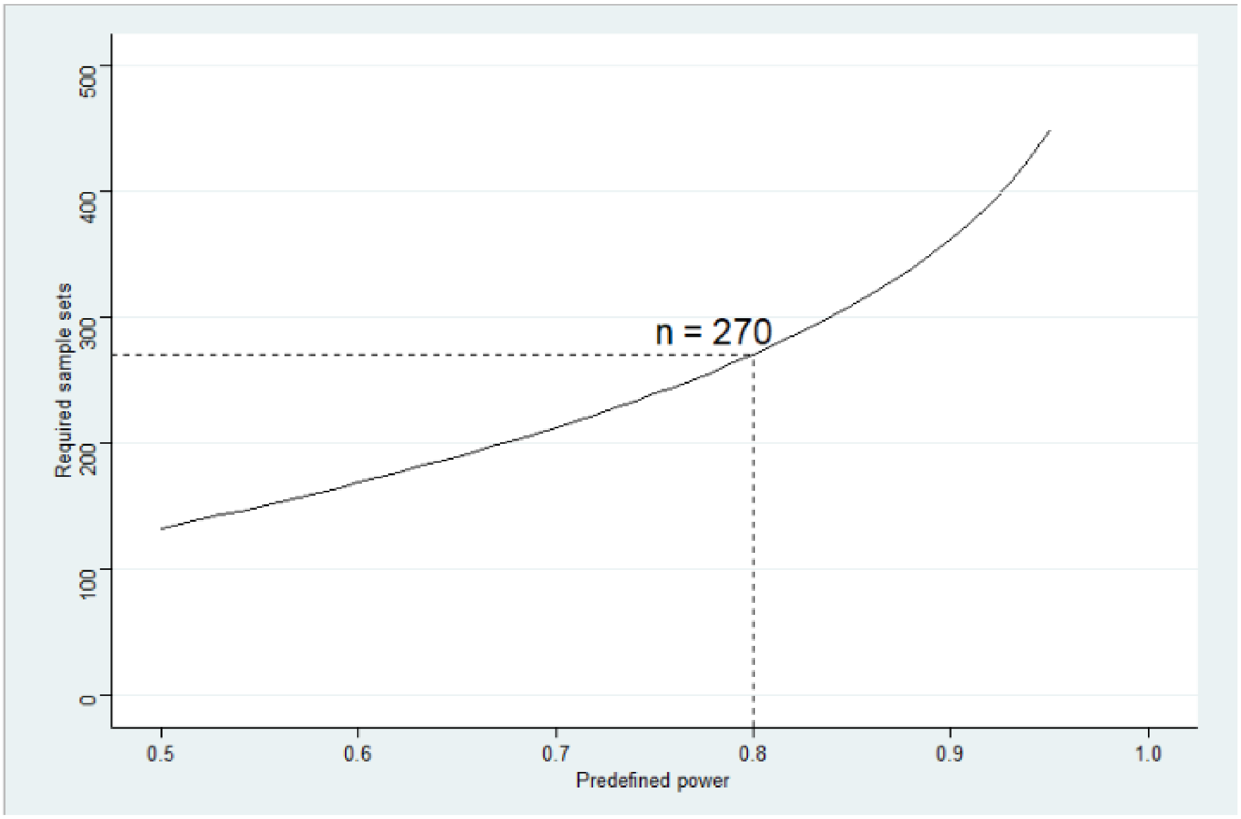

Our preliminary result showed that the adjusted odds ratio for effectiveness after the second dose BNT162b2 for adolescents was 0.68. Given that the prevalence of the extended dose was 55% of the Hong Kong population aged less than 18 years old, matched sets (1 case and 4 controls) ranging from 133 to 448 were required to achieve 60% to 95% power to detect the odds ratios of 0.68 at 0.05 significance level. With a power of 80%, the required sample sets are 270 (270 cases and 1080 controls). The line indicates the required sample sets plotted for power from 0.50 to 0.95 by 0.05. The sample size calculation was conducted using the R package “powerSurvEpi”.

Title: *Effectiveness of BNT162b2 after extending the primary-series dosing interval in children and adolescents aged 5-17*
Journal: *Nature Communications*

Supplementary Table 1. Characteristics in two case-control studies for Covid-19 infection and Covid-19 hospitalization stratified by case and exposure status

	**Covid-19 infection**					
	**Controls**			**Cases**		
	**Regular dosing interval N = 16059**	**Extended dosing interval N = 5518**	**SMD**	**Regular dosing interval N = 4148**	**Extended dosing interval N = 1248**	**SMD**
Onset time [days, median (25-75 percentile)]	201.00 [183.00, 219.00]	51.00 [19.00, 106.00]	2.562	198.00 [180.00, 215.00]	67.50 [26.00, 118.00]	2.404
Age [mean (SD)]	15.40 (1.27)	14.09 (2.52)	0.656	15.38 (1.28)	14.00 (2.61)	0.670
Sex (male ,%)	8602 (53.6)	2982 (54.0)	0.010	2194 (52.9)	703 (56.3)	0.069
Pre-existing comorbidities (%)						
Asthma	183 (1.1)	75 (1.4)	0.020	73 (1.8)	23 (1.8)	0.006
Diabetes	22 (0.1)	13 (0.2)	0.023	6 (0.1)	2 (0.2)	0.004
Epilepsy	97 (0.6)	63 (1.1)	0.058	37 (0.9)	18 (1.4)	0.051
The use of immunosuppressants within 90 days before index date (%)	5 (0.0)	14 (0.3)	0.059	0 (0.0)	3 (0.2)	0.069
	**Covid-19 hospitalization**					
	**Controls**			**Cases**		
	**Regular dosing interval N = 520**	**Extended dosing interval N = 288**	**SMD**	**Regular dosing interval N = 133**	**Extended dosing interval N = 69**	**SMD**
Onset time [days, median (25-75 percentile)]	186.00 [163.00, 212.50]	71.00 [34.75, 111.50]	2.093	188.00 [165.00, 207.00]	88.00 [57.00, 112.00]	2.304
Age [mean (SD)]	15.41 (1.30)	13.38 (2.92)	0.901	15.35 (1.33)	13.42 (3.02)	0.825
Sex (male ,%)	265 (51.0)	155 (53.8)	0.057	67 (50.4)	38 (55.1)	0.094
Pre-existing comorbidities (%)						
Asthma	9 (1.7)	2 (0.7)	0.095	5 (3.8)	0 (0.0)	0.280
Diabetes	1 (0.2)	0 (0.0)	0.062	0 (0.0)	0 (0.0)	<0.001
Epilepsy	8 (1.5)	5 (1.7)	0.016	7 (5.3)	3 (4.3)	0.043
The use of immunosuppressants within 90 days before index date (%)	0 (0.0)	0 (0.0)	<0.001	0 (0.0)	2 (2.9)	0.244

Abbreviation: standard mean difference (SMD), Standard Deviation (SD)

Title: *Effectiveness of BNT162b2 after extending the primary-series dosing interval in children and adolescents aged 5-17*
Journal: *Nature Communications*

Supplementary Table 2. The number of participants in each age strata after matching

**Age**	**Covid-19 infection**		**Covid-19 related hospitalization**	
	**Control**	**Cases**	**Control**	**Cases**
3	0	0	0	0
4	0	0	0	0
5	20	5	4	1
6	73	19	4	1
7	104	26	8	2
8	77	20	16	4
9	124	31	4	1
10	144	36	8	2
11	148	37	16	4
12	219	55	16	4
13	2032	508	92	23
14	4340	1085	148	37
15	4560	1140	172	43
16	4708	1177	132	33
17	5028	1257	188	47
Sum	21577	5396	808	202
